# Characteristics and drivers of plant C, N, and P stoichiometry in Northern Tibetan Plateau grassland

**DOI:** 10.3389/fpls.2023.1092872

**Published:** 2023-04-06

**Authors:** Wei Wang, Jiamin Zhao, Zhen Xing, Xiangtao Wang

**Affiliations:** ^1^ College of Resources and Environmental Sciences, Tibet Agriculture and Animal Husbandry University, Nyingchi, Tibet, China; ^2^ Key Laboratory of Forest Ecology in Tibet, Ministry of Education, Xizang Agriculture and Animal Husbandry College, Nyingchi, Tibet, China; ^3^ Tibet Agricultural and Animal Husbandry University, College of Animal Science, Nyingchi, Tibet, China

**Keywords:** C, N, and P stoichiometry, plant and soil, grassland type, influence factors, North Tibetan Plateau

## Abstract

Understanding vegetation C, N, and P stoichiometry helps us not only to evaluate biogeochemical cycles and ecosystem functions but also to predict the potential impact of environmental change on ecosystem processes. The foliar C, N, and P stoichiometry in Northern Tibetan grasslands, especially the controlling factors, has been highlighted in recent years. In this study, we have collected 340 plant samples and 162 soil samples from 54 plots in three grassland types, with the purpose of evaluating the foliar C, N, and P stoichiometry and underlying control factors in three grassland types along a 1,500-km east-to-west transect in the Northern Tibetan Plateau. Our results indicated that the averaged foliar C, N, and P concentrations were 425.9 ± 15.8, 403.4 ± 22.2, and 420.7 ± 30.7 g kg^−1^; 21.7 ± 2.9, 19.0 ± 2.3, and 21.7 ± 5.2 g kg^−1^; and 1.71 ± 0.29, 1.19 ± 0.16, and 1.59 ± 0.6 g kg^−1^ in the alpine meadow (AM), alpine steppe (AS), and desert steppe (DS) ecosystems, respectively. The foliar C and N ratios were comparable, with values of 19.8 ± 2.8, 20.6 ± 1.9, and 19.9 ± 5.8 in the AM, AS, and DS ecosystems, respectively. Both the C/P and N/P ratios are the lowest in the AM ecosystem, with values of 252.2 ± 32.6 and 12.8 ± 1.3, respectively, whereas the highest values of 347.3 ± 57.0 and 16.2 ± 3.2 were obtained in the AS ecosystem. In contrast, the soil C, N, C/P, and N/P values decreased from the AM to DS ecosystem. Across the whole transects, leaf C, N, and P stoichiometry showed no obvious trend, but soil C and N concentrations showed an increasing trend, and soil P concentrations showed a decreasing trend with the increasing longitude. Based on the general linear model analysis, the vegetation type was the dominant factor controlling the leaf C, N, and P stoichiometry, accounting for 42.8% for leaf C, 45.1% for leaf N, 35.2% for leaf P, 52.9% for leaf C/N, 39.6% for leaf C/P, and 48.0% for leaf N/P; the soil nutrients and climate have relatively low importance. In conclusion, our results supported that vegetation type, rather than climatic variation and soil nutrients, are the major determinants of north Tibet grassland leaf stoichiometry.

## 1 Introduction

Elemental stoichiometry can reflect the interactions between plants and soil and link biogeochemical cycles to physiological constraints (Sterner and Elser, 2002). Carbon (C), nitrogen (N), and phosphorus (P) are generally considered to be key macronutrients of all organisms that play vital roles in metabolism and ecosystem nutrient cycling (Elser et al., 2000; Elser et al., 2010). The shifts in C, N, and P contents and their ratios are considered to be crucial because they not only are closely related to the plant growth rate and photosynthesis but also act as indicators of the whole ecosystem limited by N or P (Hall et al., 2005; Wieder et al., 2015). Thus, understanding the foliar C, N, and P stoichiometry can help to predict the potential effects of environmental change on ecosystem processes, such as atmospheric N and P deposition and grassland degradation (Wang et al., 2017; Wang XG. et al., 2020; Yue et al., 2017).

Over the past decades, increasing attention has been paid to the C, N, and P stoichiometry in plants and soils at different scales (Reich and Oleksyn, 2004; Han et al., 2005; Tao et al., 2016; Wang XG. et al., 2020; Liu et al., 2021). It was proven that elemental stoichiometry is correlated with geographic and climatic variables (Wu et al., 2012; Sardans et al., 2015; Fan et al., 2016; Hu et al., 2017; Gong et al., 2018; Zhang et al., 2018; Wang et al., 2021). Many studies have been conducted to explain factors influencing plant ecological stoichiometry. More and more biotic and abiotic factors had been used to explain the changes in leaf C, N, and P stoichiometry, such as degradation light (Zhu et al., 2020), solar radiation (Sun et al., 2019), elevation (Zhao et al., 2018), and slope aspect (Cao et al., 2020). The balance and stoichiometric ratios of C, N, and P in vegetation and soil can be affected by drought (Delgado-Baquerizo et al., 2013; Wang et al., 2019, Wang Y. et al., 2020). Drought has direct effects on plant physiological characteristics. On the one hand, increasing aridity might decrease the plant nutrient uptake and transport (Bista et al., 2018); on the other hand, plant nutrient concentrations might increase to maintain the physiological metabolism under drought conditions (Griffiths and Parry, 2002). However, it had been founded that the climatic factors exerted limited influence on leaf N and P concentrations (He et al., 2008). The reason might be that the variation of leaf N and P largely depends on plant species and that plant nutrients largely depend on the identity of the species experiencing drought stress (Bertiller et al., 2005).

Several hypotheses have been proposed to explain the plant’s ecological stoichiometry (Tian et al., 2021). The temperature–biogeochemistry, temperature–plant physiology, and soil substrate age hypotheses have been used to explain the spatial variations in plant ecological stoichiometry (Reich & Oleksyn, 2004). Considering that the ecosystems in Tibet are at high altitudes, experience low temperatures, and have young soil ages, the foliar N and P concentrations in these areas might be high. The species composition hypothesis suggests that differences in species or life type composition affect the spatial patterns of the stoichiometric characteristics of plant leaves (Reich & Oleksyn, 2004). A similar phenomenon was observed east of the Tibetan Plateau (Zhao et al., 2018). Although the plant C, N, and P stoichiometry has been investigated in different regions across Qinghai–Tibet (Hong et al., 2014; Cai et al., 2019; Wang Y. et al., 2020), knowledge of the variations in the plant C, N, and P stoichiometry in different grassland ecosystems in north Tibet as well as the influencing factors remains limited.

The Tibetan Plateau is highly sensitive to climate change and is mainly covered by grassland (Tan et al., 2010). In north Tibet grassland, plant species declined significantly coupled with annual precipitation amounts from east to west. There are three types of grassland in those areas: alpine meadow (AM), alpine steppe (AS), and desert steppe (DS). On the one hand, grassland degradation is widespread here caused by climate change (Wang Y. et al., 2020; Shi et al., 2022); on the other hand, the restoration and treatment of degraded grasslands were widely carried out in those areas (Chen et al., 2020). This situation means that the shifting and changing of different grassland types in the Qinghai–Tibet Plateau are occurring nowadays. However, the differences between the foliar C, N, and P stoichiometry and control factors between different grasslands in this area remain unexplored. This may result in some biases in the prediction of the biogeochemical cycles under global change in the Qinghai–Tibet Plateau. In this study, we investigated the C, N, and P concentrations and C:N:P ratios of plants as well as topsoil (0–20 cm) across Northern Tibetan Plateau grassland transects. We aimed to answer the following questions: 1) What are the characteristics of the C, N, and P stoichiometry in three typical grassland ecosystems across North Tibetan grassland transects? (2) What are the control factors associated with the C:N:P stoichiometry in these ecosystems? (3) Are the control factors of stoichiometric characteristics in different grasslands consistent?

## 2 Materials and methods

### 2.1 Study area

**Figure 1:**
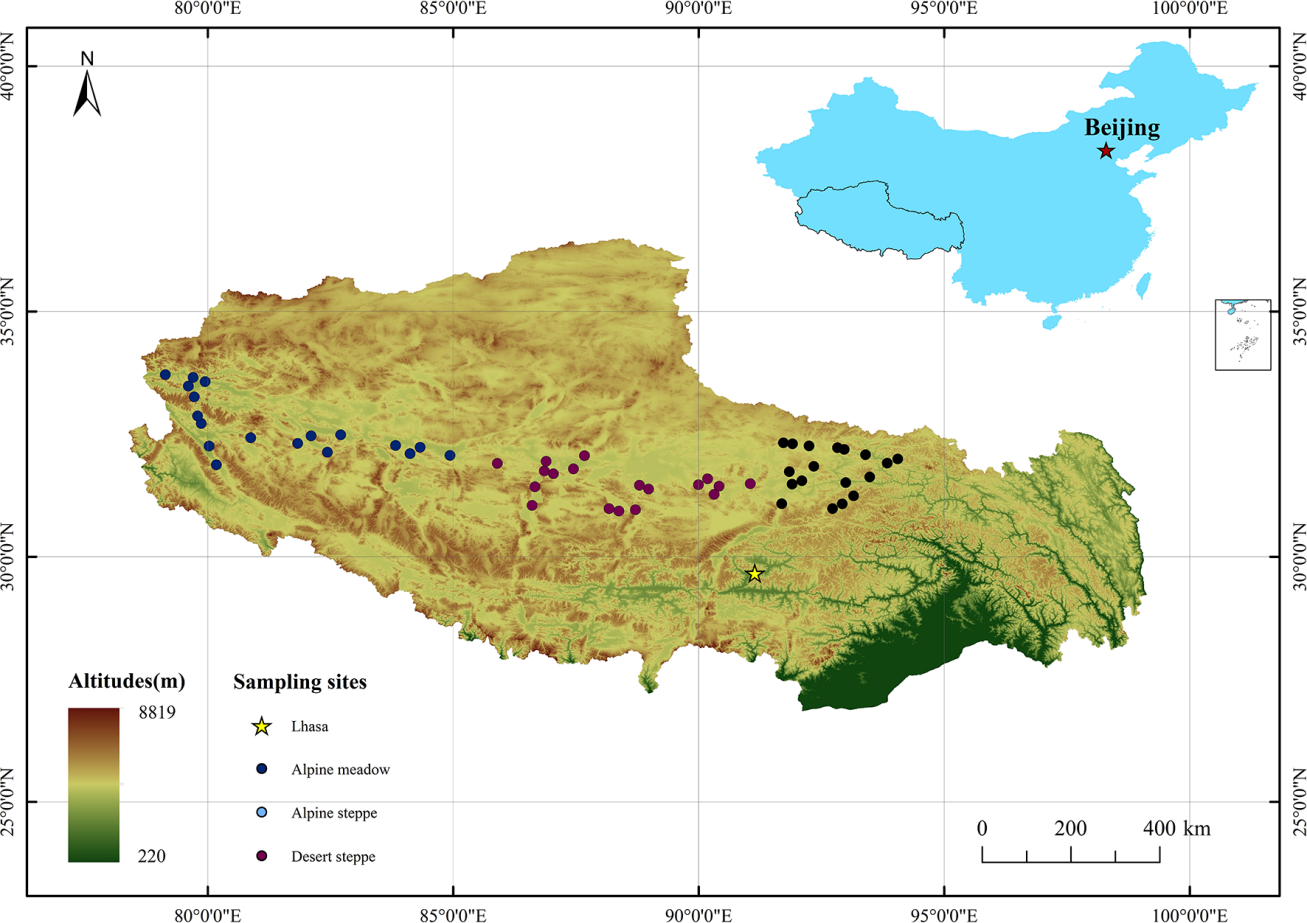
Location of the sampling sites.

This study was conducted along a 1,500-km east-to-west transect in the North Tibetan Plateau (30°–34°N, 79°–95°E). This transect contains three typical types of grassland: AM, AS, and DS. We classified the grassland types based on the dominant species. The dominant species in AM and AS are *Kobresia* and grass (such as *Stipa purpurea* and *Stipa capillacea*), respectively. The dominant species in DS are *Christolea crassifolia*, *Ajania fruticulosa*, *Krascheninnikovia ceratoides*, and *Stipa glareosa*. Eighteen sampling sites were selected for each grassland type (**Figure 1**).

### 2.2 Plant and soil sampling

Plant samples were harvested by hand during the near-peak biomass period in August 2020. The samples were obtained from flat areas with uniform vegetation. Foliar samples were collected according to previous studies (Wang et al., 2022). During the leaf collection period, only mature leaves free from pests and diseases were harvested and placed in an envelope. Each plant species was collected over the largest possible area. For each species, leaves from a minimum of 50 individual plants were collected. In addition, each species with a leaf fresh weight of not less than 500 g was harvested. Rare species were not harvested because of their low numbers and biomass. In total, 340 plant samples were collected from 54 sampling sites.

Five replicates of 0–20-cm soil columns were collected and mixed into one sample using a stainless steel boring auger. Three soil samples were collected from each sampling site. In total, 162 soil samples were collected from 54 sampling sites. The spatial geographical coordinates of each site were obtained using GPS (CaiTu C86, HuaChengBeiDou, China). The collected samples were placed in plastic bags and taken to the laboratory.

### 2.3 Sample preparation and chemical analyses

In the laboratory, samples of plant leaves were washed with tap water to remove dust and impurities, rinsed with ionized water, oven-dried at 120°C for 1 h, and then oven-dried at 70°C until the mass was constant. Dry leaves were ground using a ball mill (vibration ball mill GT300, Beijing Grinder Instrument Co., Ltd., Beijing, China) and stored in a Ziploc bag prior to the C, N, and P analyses. All soil samples were air-dried in the laboratory. Before the analysis, soil samples were sieved through a 0.5-mm sieve to remove stones, litter, and plant roots and then stored in a Ziploc prior to the C, N, and P analyses.

The C, N, and P determination methods for both plants and soil followed those specified by Bao (2000). Briefly, the organic C (C) content was analyzed using the potassium dichromate–sulfuric acid oxidation method. The total N (TN) content was determined using Kjeldahl digestion. The total P (TP) content was colorimetrically determined using the ammonium molybdate method.

### 2.4 Statistical analyses

All plant samples from different grasslands were used for C, N, and P distribution frequency calculations. First, the geometric means of leaf C:N:P stoichiometry were calculated for all species at a sampling site. Then, the arithmetic means of leaf C:N:P stoichiometry were calculated based on previously calculated geometric means in 18 sampling sites from different grasslands.

General linear models (GLMs) were used for ANOVA according to previous studies (He et al., 2008). Explanatory terms were related to climatic variables ((mean annual precipitation (MAP), mean annual temperature (MAT), soil nutrients (soil C concentrations (SC), soil N concentrations (SN), and soil P concentrations (SP)), and soil available nutrient (soil available N concentration (AN) and soil available P concentrations (AP)), and species. In addition, leaf traits were log-10 transformed prior to analysis. MAT data were obtained from http://data.tpdc.ac.cn/zh-hans/data/ (Du and Yi, 2019). The MAP was obtained from http://data.tpdc.ac.cn/zh-hans/data/ (Fang, 2019).

Spearman’s correlation analysis was used to evaluate the correlations between soil, plant nutrients, and climate factors. Statistical analyses were performed using SPSS 26.0 (SPSS Inc., Chicago, IL, USA), Origin 2019b (OriginLab Co., Northampton, MA, USA).

## 3 Results

### 3.1 Variations of leaf C, N, P, and C:N:P ratios in the North Tibetan Plateau

**Figure 2:**
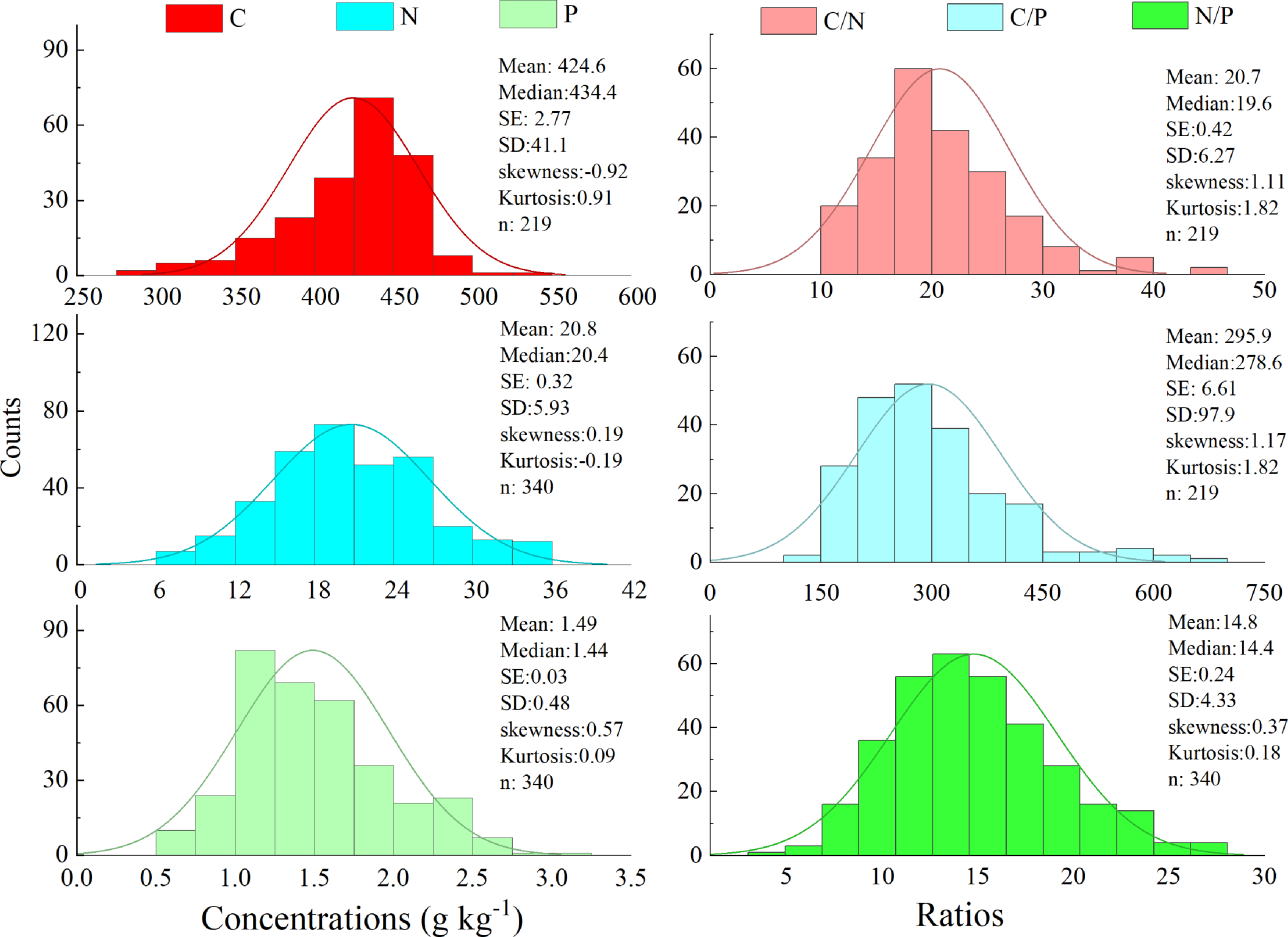
Frequency of the foliar C, N, and P stoichiometry in study area.

The leaf C, N, and P contents of all species in different grasslands ranged at 292.8–536.4, 6.03–35.9, and 0.42–2.98 g kg^−1^ in this work, respectively. The element ratios varied greatly, with a range of 10.9–45.6 for C:N, 176.3–696.4 for C:P, and 4.83–27.2 for N:P in this work (**Figure 2**).

### 3.2 Comparison of the C, N, and P concentrations in leaves and soil in different grasslands

**Figure 3:**
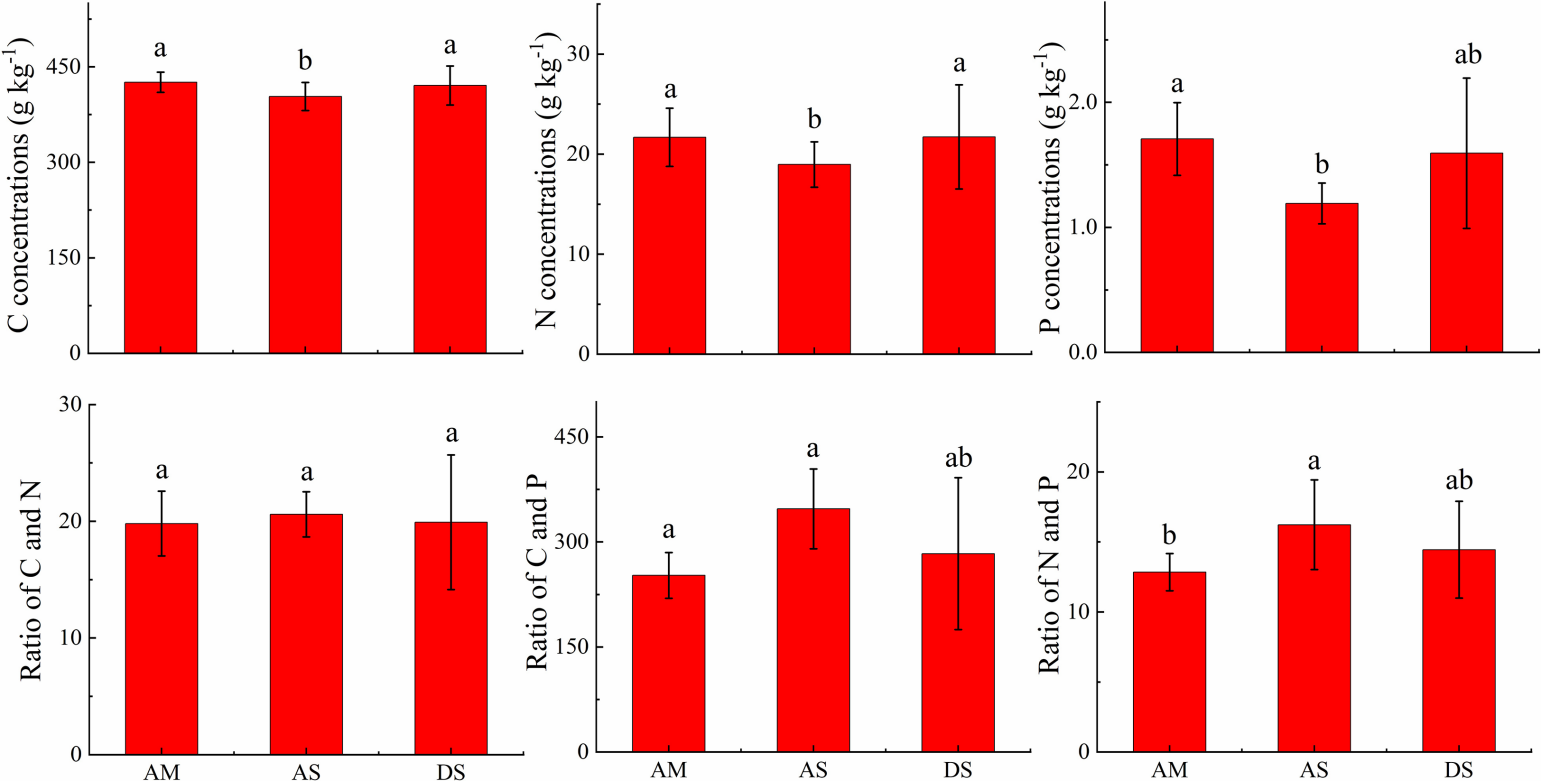
Comparison of foliar C, N, and P stoichiometry in different grassland types. Different letters above the column means the differences was significant at 0.05 level.

The foliar C concentrations in AM, AS, and DS were 425.9 ± 15.8, 403.4 ± 22.2, and 420.7 ± 30.7 g kg^−1^, respectively; the foliar N concentrations were 21.7 ± 2.9, 19.0 ± 2.3, and 21.7 ± 5.2 g kg^−1^, respectively; and the foliar P concentrations were 1.71 ± 0.29, 1.19 ± 0.16, and 1.59 ± 0.6 g kg^−1^, respectively. The foliar C, N, and P concentrations in the AS were the lowest. The foliar C and N ratios were comparable, with values of 19.8 ± 2.8, 20.6 ± 1.9, and 19.9 ± 5.8 in the AM, AS, and DS, respectively. The foliar C and P ratio in the AS is the highest, with a value of 347.3 ± 57.0. The foliar N and P ratio in the AS is the highest, with a value of 16.2 ± 3.2 (**Figure 3**).

**Figure 4:**
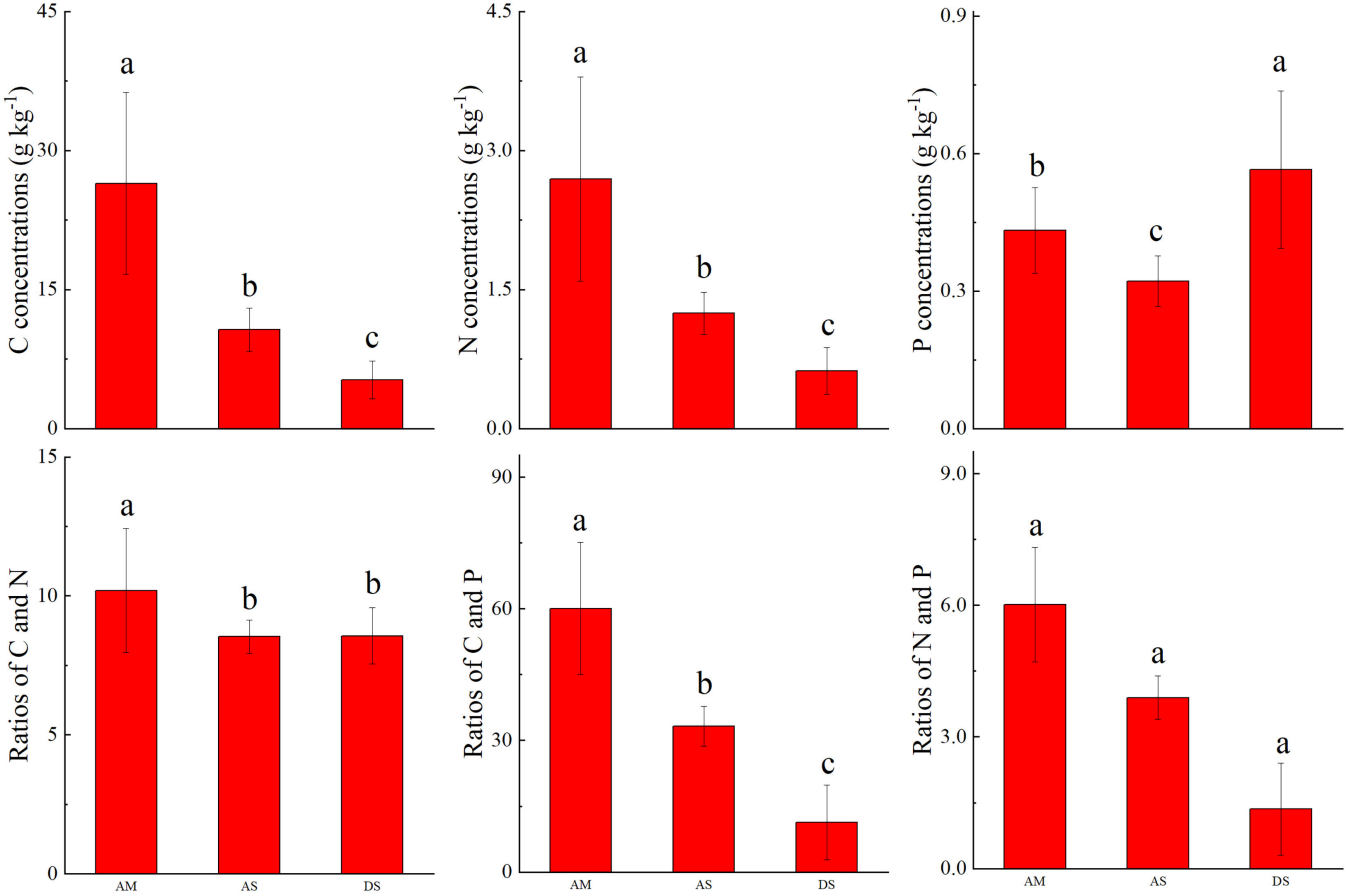
Comparison of soil C, N, and P stoichiometry in different grassland types. Different letters above the column means the differences was significant at 0.05 level.

The soil C concentrations were 26.5 ± 9.8, 10.7 ± 2.3, and 5.26 ± 2.05 g kg^−1^, whereas the soil N concentrations were 2.69 ± 1.1, 1.24 ± 0.23, and 0.62 ± 0.25 g kg^−1^ in the AM, AS, and DS, respectively. In addition, both soil C and N concentrations were significantly lower in the AS. The soil P concentrations were 0.43 ± 0.09, 0.32 ± 0.06, and 0.56 ± 0.17 g kg^−1^ in the AM, AS, and DS ecosystems, respectively. The soil C and N ratio was the highest in the AM, with a value of 10.2 ± 2.2. The values in the AS and DS ecosystems were comparable. The soil C and P ratio was the highest in the AM, with a value of 60.1 ± 15.0. The foliar N and P ratio was the highest in the AS, with a value of 6.01 ± 1.3 (**Figure 4**).

**Figure 5:**
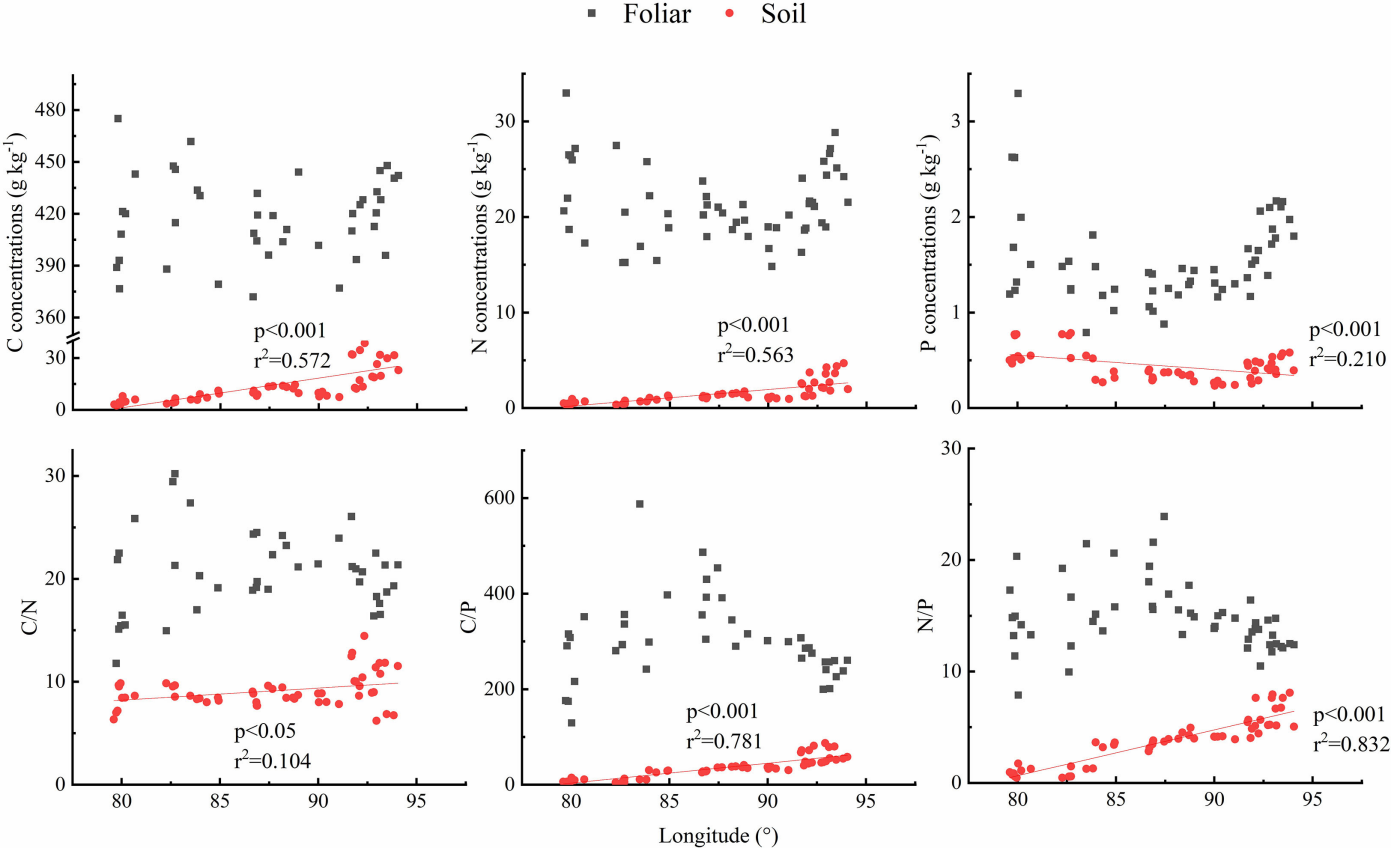
Longitude pattern of foliar and soil C, N, and P stoichiometry across north Tibet grassland.

Considering the grassland types were changed with longitude changing, the longitude patterns of leaf and soil C, N, and P stoichiometry were analyzed. Results showed that no obvious trend was observed of the foliar C, N, and P concentrations and foliar C/N, C/P, and N/P, yet soil C and N concentrations showed an increasing trend, and soil P concentrations showed a decreasing trend with the increasing of longitude, soil C/P. In addition, N/P dramatically increased with the increase in longitude (**Figure 5**).

### 3.3 Relationships between foliar C, N, and P concentrations and soil nutrients in different grasslands

In the AM, foliar C concentrations (C) were positively correlated with the soil available N (AN) at 0.05, and the soil C, N, and P concentrations were significantly positive (**Table S2**). In the AS, C showed significant positive correlations with AN, foliar N concentrations (N) showed positive correlations with soil P concentrations (SP), and soil C, N, and P concentrations were significantly positive (**Table S3**). In the DS, C showed a significantly positive relationship with AN, and N showed a positive correlation with SN at a 0.05 level. Soil C was significantly positively correlated with soil N, but no correlation was observed between soil C and soil P (**Table S4**).

### 3.4 Relative effects of the vegetation type, soil, and climate

The results of the GLM showed that species (vegetation type) were the dominant factors of the six leaf C:N:P traits, accounting for 42.8% for leaf C, 45.1% for leaf N, 35.2% for leaf P, 52.9% for leaf C:N, 39.6% for leaf C:P, and 48.0% for leaf N:P. The contributions of other factors (MAP, MAT, SC, SN, SP, AN, and AP) to leaf C:N:P traits were all limited (**Table 1**).

**Table 1:** Summary of the general linear models for the effect of environmental variations.

		C				N				P				C/N				C/P				N/P			
	df	MS	F	P	%SS	MS	F	P	%SS	MS	F	P	%SS	MS	F	P	%SS	MS	F	P	%SS	MS	F	P	%SS
MAP	7	0.000	0.220	0.803	0.15	0.050	6.832	0.000	5.86	0.034	4.067	0.000	3.04	0.001	0.125	0.882	0.05	0.019	2.046	0.136	0.82	0.014	1.076	0.381	1.32
MAT	7	0.001	0.378	0.769	0.38	0.014	1.892	0.074	1.62	0.030	3.590	0.001	2.69	0.011	1.692	0.175	1.02	0.008	0.802	0.496	0.48	0.013	0.992	0.439	1.22
SC	3	0.001	0.359	0.551	0.12	0.005	0.703	0.551	0.26	0.011	1.305	0.274	0.42	0.002	0.252	0.617	0.05	0.004	0.424	0.517	0.09	0.009	0.689	0.560	0.36
SN	3	0.001	0.369	0.551	0.76	0.002	0.316	0.814	0.12	0.001	0.062	0.980	0.02	0.001	0.083	0.776	0.79	0.004	0.236	0.634	0.31	0.001	0.079	0.971	0.04
SP	3	0.000	0.037	0.848	0.01	0.010	1.328	0.267	0.49	0.008	0.998	0.395	0.32	0.004	0.629	0.430	0.13	0.002	0.239	0.626	0.05	0.018	1.434	0.235	0.75
AP	4	0.001	0.773	0.382	0.26	0.030	4.103	0.003	2.01	0.043	5.196	0.001	2.22	0.001	0.144	0.705	0.03	0.052	5.486	0.022	1.10	0.019	1.460	0.216	1.02
AN	5	0.002	1.707	0.188	1.16	0.013	1.729	0.130	1.06	0.014	1.721	0.132	0.92	0.001	0.098	0.907	0.04	0.003	0.266	0.767	0.11	0.006	0.432	0.826	0.38
Species	96	0.002	1.756	0.006	42.8	0.028	3.830	0.000	45.1	0.029	3.432	0.000	35.2	0.023	3.641	0.000	52.9	0.026	2.736	0.000	39.6	0.036	2.852	0.000	48.0
Residuals					54.4				43.5				55.2				45.0				57.5				46.9

Leaf N, P, and N/P were log-10 transformed prior to analysis.

MAP, mean annual precipitation; MAT, mean annual temperature; SC, soil C concentrations; SN, soil N concentrations; SP, soil P concentrations; AP, soil available P concentrations; AN, soil available N concentrations; MS, mean sum of square; %SS, percentage of sum of squares explained.

## 4 Discussion

### 4.1 Comparison of the C, N, and P concentrations among different grasslands

Foliar C concentrations have a significantly higher stoichiometric homeostasis than other nutrients (Zhang et al., 2020). Similar results were obtained in this study (**Figure 3**). The standard deviations of foliar C concentrations in the three ecosystems were low. The average foliar C concentrations in the AM and DS ecosystems were comparable, whereas they were slightly lower in the AS ecosystem (**Figure 3**). In addition, the foliar C concentrations ranged from 405.2 to 424.5 g kg^−1^, which is close to the result for Northern Tibet reported by Ma et al. (2019).

Foliar N and P concentrations have been widely investigated in China’s grasslands. Han et al. (2005) reported that terrestrial ecosystems were P-poor based on the plant N and P stoichiometry. As shown in **Table 2**, compared with the average foliar N and P concentrations in global flora, the foliar N concentrations were at a high level and foliar P concentrations were generally at a low level in most studies carried out in China. Similar results were observed in this study; the foliar N concentrations were comparable to the average global values, whereas the foliar P concentrations of all three types of grassland were lower than the average global values (**Table 2**).

**Table 2:** Comparison of foliar C, N, and P concentrations among different grasslands.

Sites	Vegetation types	C (g kg^−1^)	N (g kg^−1^)	P (g kg^−1^)	References
Global flora	All		20.6	1.99	Elser et al., 2000
Global flora	All		20.1	1.80	Reich and Oleksyn, 2004
Mexico	Desert	427.5	23.0	1.60	Castellanos et al., 2018
England	Grassland		27.8	2.72	Thompson et al., 1997
China	All		20.2	1.46	Han et al., 2005
China	Desert		**24.5**	1.74	Li et al., 2010
China	Grassland and desert		**33.8**	2.30	He et al., 2006
China	Grassland		**29.0**	1.90	
China	Grassland	463.8	19.9	1.31	Yu et al., 2017a
North China	Grassland	392.1–425.9	8.5–16.3	1.03–2.25	Wang et al., 2021
Loess Plateau	Desert	434.4	18.9	1.27	Yang et al., 2018
Qilian Mountains	Desert		**28.4**	1.78	Xu et al., 2015
	Grassland		**23.1**	1.55	
	Alpine meadow		**26.4**	2.58	
Xinjiang	Desert		**30.8**	1.77	Ye et al., 2016
Inner Mongolian Plateau	Grassland		19.4	1.33	Yu et al., 2017b
Inner Mongolia	Grassland	460.8	17.0	1.11	Fan et al., 2016
Qinghai–Tibet	Grassland	464.9	21.0	1.45	Fan et al., 2016
Qinghai–Tibet	Grassland		21.0	1.48	Yu et al., 2017b
Tibetan Plateau	Alpine steppe		**29.7**	1.80	He et al., 2008
Tibetan Plateau	Grassland		**23.5**	1.90	
Tibetan Plateau	Meadow steppe		**24.2**	1.60	
East Tibetan Plateau	Grassland		**25.3**	1.70	Sun et al., 2019
Northern Tibetan Plateau	Alpine steppe	410.5	**24.7**	1.40	Ma et al., 2019
North Tibet	Alpine steppe		**23.2**	1.38	Hong et al., 2014
North Tibet	Tibetan Plateau grassland		**23.5**	1.90	Yang et al., 2010
North Tibet	Alpine meadow	424.5	22.5	1.75	This study
	Alpine steppe	405.2	19.7	1.25	
	Desert steppe	423.2	22.0	1.64	

Bold values means the foliar N conceontrations were higher than those in this work.

In Tibetan grassland ecosystems, foliar N concentrations ranged from 21.0 to 29.7 g kg^−1^, whereas foliar P concentrations ranged from 1.38 to 1.90 g kg^−1^ (**Table 2**). Additionally, foliar P concentrations varied considerably in the three grassland types, with the value being higher in AM and lower in AS. The high foliar P concentrations in the AM ecosystem can be explained by the amount of precipitation. P is primarily derived from the weathering of soil inorganic components and the degradation of organic matter (Aerts and Chapin, 1999). Precipitation, which may amplify the P availability in soil by facilitating litter decomposition in arid regions, is at a high level in AM ecosystems (**Table S1**). Similar results were observed in the Qinglian Mountains (Xu et al., 2015).

For the same transect, He et al. (2006; 2008). reported that variations in the foliar N and P concentrations were mainly influenced by geographic and between-species variations. The results of this study show that the vegetation type was the most important control factor (**Table 1**). Furthermore, low foliar P concentrations and high C/P ratios were observed in the AS ecosystem in this work (**Figure 4**). The reason might be that the leaf nitrogen and phosphorus stoichiometry commonly differ at the family level and that low foliar N and P concentrations have existed in both Gramineae and Cyperaceous (Tian et al., 2018). Meanwhile, the dominant species were Gramineae and Cyperaceous in the AS ecosystem, and the foliar N and P concentrations were at a low level in both in this work (Appendix Figure 1).

### 4.2 Relationships of the foliar C, N, and P concentrations, soil nutrients, and climate factors

C concentrations were relatively weakly correlated with other nutrients in the same organ (Zhang et al., 2020). In this study, the foliar C concentration was not correlated with the foliar N and P contents in the three grassland types. The reason might be that foliar C concentrations are relatively stable in the leaf (**Figure 3**) (Zhang et al., 2020), but foliar N and P concentrations varied largely due to biological or abiotic factors (**Table 1**). For example, plant nutrient concentrations might increase to maintain the physiological metabolism under drought conditions (Griffiths and Parry, 2002). In this work, the foliar N and P concentrations were high in the DS ecosystem than those in the AS ecosystem, whereas foliar C concentrations were comparable in those two ecosystems (**Figure 3**). As a result, the correlation between leaf C and N concentrations was poor, as well as foliar C and P concentrations. A similar result was observed for the Hexi Corridor (Zhang et al., 2020).

Generally, the leaf N and P concentrations are positively correlated in field environments (Gusewell, 2004). Similar results were obtained in this study (Appendix Figure 2). However, a correlation between the foliar N and P concentrations was not observed in the AS ecosystem (**Table S3**). The reason might be that plant N and P could be more strongly coupled in humid conditions than in arid environments across alpine grasslands (Zhou et al., 2020).

The foliar C of AN showed positive correlations in all three grasslands in this study. The reason might be that these ecosystems are limited by N (Xie et al., 2020). Interestingly, a negative correlation was observed between foliar and soil N in the DS (**Table S4**). This might be because the soil N concentration is mainly influenced by N-fixing microbes (Houlton et al., 2018). In the DS ecosystem, soil N is relatively low because of poor environmental conditions (**Figure 4**, **Table S1**). However, in drought environments, plants tend to increase nutrient concentrations to maintain their physiological metabolism. Consequently, a negative correlation between foliar N and soil N was observed in the drought environment.

There is no doubt that the Qinghai–Tibet Plateau has a high altitude and low temperature; consequently, its soil is young and rich in P (Wang et al., 2013). As MAP increases, soil AP may show an increasing trend because precipitation amount can amplify the P availability (Aerts and Chapin, 1999). In this study, leaf P concentrations were positively correlated to MAP (**Table S5**). Meanwhile, vegetations tend to increase the foliar nutrient concentrations in low-temperature environments, and both leaf N and P concentrations show increasing trends as temperature increased when the annual temperature is below 5°C (Reich and Oleksyn, 2004). Leaf N concentrations were positively correlated to MAT in this work.

### 4.3 Foliar N and P stoichiometry and ecosystem limitations in north Tibet grassland

The foliar N and P concentrations vary greatly depending on several factors (e.g., climate, soil nutrient pool, and vegetation types). The results of this study show that the foliar C, N, and P concentrations decreased significantly from AM to AS. This can be explained by the biogeochemical hypothesis, which states that the concentrations of N and P in plant tissues are controlled by the availability of soil N and P; thus, the concentrations of N and P in plant tissues are highly correlated with those in the soil (McGroddy et al., 2004; Reich and Oleksyn, 2004). As shown in **Figure 2**, the soil N and P concentrations decreased dramatically from AM to AS. Furthermore, the leaf P concentrations increased with the precipitation because the increase in the precipitation may amplify the P availability in soil by facilitating the decomposition of litter in arid regions (Aerts and Chapin, 1999). The precipitation amounts were higher in the AM than in AS in the research areas (**Table S1**). The vegetation type was the dominant factor affecting foliar C, N, and P concentrations (**Table 1**), which differed in the two grasslands. The foliar C, N, and P concentrations increase from AS to DS. The soil N and P concentrations significantly decrease from AS to DS. This can be explained by the plant physiology hypothesis, which refers to the increase in the foliar N and P concentrations to offset the decrease in the plant metabolic rate when the ambient temperature decreases (Reich and Oleksyn, 2004) as well as in arid environments (Cunningham et al., 1999; Wright et al., 2005).

The C, N, and P stoichiometry is commonly used to evaluate nutrient limitations in ecosystems (Liu et al., 2021; Wang et al., 2021; Zhang et al., 2021). In general, high C:N and low N:P ratios were considered to be N-limited, whereas high C:P and N:P ratios were considered to be P-limited. The C:P and N:P ratios were the highest in the three grasslands because the foliar P concentrations in the AS ecosystem were low. This indicates that a P limitation might exist in the AS ecosystem of the Qinghai–Tibet Plateau. In addition, Koerselman (1996) suggested that the leaf N:P ratio can be used to reveal N limitations (N:P ratio < 14) or P limitations (N:P ratio > 16) in the ecosystem. In this study, the foliar N and P ratios were determined to be 13.1, 16.8, and 14.8 in the AM, AS, and DS ecosystems, respectively. The leaf N:P ratio in the AS ecosystem is above 16, suggesting that those areas might be restricted by P, whereas the ratio was below 14 in the AM ecosystem, suggesting that those areas might be restricted by N in the Northern Tibetan Plateau grassland.

### 4.4 Implications and uncertain analysis

Our result agreed that the between-species variation, rather than climatic variation, is the major determinant of grassland foliar stoichiometry at the biome level (He et al., 2008). This difference in response of leaf C, N, and P stoichiometry to environmental factors caused by vegetation type changes may be the main reason for the weak interpretation of environmental factors to leaf C, N, and P stoichiometry in the whole Northern Tibet transects.

Although soil nutrients, MAP, and MAT were commonly used to explain the spatial variations of leaf C, N, and P stoichiometry (Zhao et al., 2018; Wang et al., 2021), in the Qinghai–Tibet Plateau, the environmental factors may be more complex, such as light, solar radiation, elevation, and drought, which are important factors affecting the leaf C, N, and P stoichiometry (Zhao et al., 2018; Sun et al., 2019; Wang et al., 2022). Not all environmental factors were examined in this study, which may result in biases in the results of this study. Therefore, it is of great significance to understand the effects of environmental factors on leaf C, N, and P stoichiometry in different communities on the Qinghai–Tibet Plateau. More comprehensive and systematic studies needed to be conducted in the future.

## 5 Conclusion

In this study, the plant and soil C, N, and P stoichiometry and its driving forces in different grassland types in Tibet were investigated. The foliar C, N, and P concentrations in AM and DS ecosystems were comparable, whereas these values were the lowest in the AS ecosystem. The foliar C and N ratios of the three types of grassland are comparable. The foliar C and P ratios are the highest and lowest in the AS and AM ecosystems, respectively. The foliar N and P ratios are the highest in the AS and the lowest in the AM. In addition, the AM ecosystem might be limited by N, and the AS ecosystem might be limited by P in Northern Tibetan grassland ecosystems. Across the whole transects, vegetation species was the dominant factor that control the leaf C, N, and P stoichiometry. Our results suggested that between-species variation, rather than climatic variation and soil nutrients, is the major determinant of north Tibet grassland leaf stoichiometry.

## Data availability statement

The raw data supporting the conclusions of this article will be made available by the authors, without undue reservation.

## Author contributions

WW designed the experiments. WW, JZ, ZX, and XW conducted the experiments. WW and ZJ made the figures. WW wrote the manuscript. All authors contributed to the article and approved the submitted version.

## Appendix Figure 1

Comparison of foliar N and P concentrations at family level.

## Appendix Figure 2

The correlation between foliar N and P in different ecosystems (**A**: AM ecosystem; **B**: AS ecosystem; **C**: DS ecosystem; **D**: all sample).
