# Supplementary material for: Characteristics and drivers of plant C, N, and P stoichiometry in Northern Tibetan Plateau grassland
Source: Front Plant Sci. 2023 Apr 6;14:1092872. doi: 10.3389/fpls.2023.1092872 (PMC10118023; doi:10.3389/fpls.2023.1092872)
Supplement: Supplementary file 1 [file DataSheet_1.docx]

| Table S1 The model data of MAT and MAP in each sampling point | | | | | | | | | |  |
| --- | --- | --- | --- | --- | --- | --- | --- | --- | --- | --- |
| Sites | N | E | MAP(mm) | MAT(°F) | Sites | N | E | MAP(mm) | MAT(°F) |  |
| 1 | 31°59′45″ | 94°3′11″ | 658.20 | 29.06 | 28 | 30°58′55″ | 88°10′24″ | 340.50 | 17.24 |  |
| 2 | 31°14′37″ | 93°9′14″ | 750.40 | 21.74 | 29 | 32°3′46″ | 87°40′17″ | 291.90 | 27.10 |  |
| 3 | 31°34′46″ | 93°7′3″ | 732.60 | 19.90 | 30 | 31°47′40″ | 87°26′55″ | 277.90 | 29.63 |  |
| 4 | 31°54′42″ | 93°50′19″ | 594.60 | 29.37 | 31 | 31°56′50″ | 86°53′26″ | 278.60 | 29.08 |  |
| 5 | 31°37′43″ | 93°28′50″ | 710.80 | 27.04 | 32 | 31°40'1″ | 86°5'20″ | 319.10 | 30.32 |  |
| 6 | 32°4'56″ | 93°23'48″ | 569.40 | 25.54 | 33 | 31°4′38″ | 86°50′56″ | 329.60 | 30.40 |  |
| 7 | 32°11′24″ | 92°57′40″ | 582.80 | 17.94 | 34 | 31°29′28″ | 86°41′37″ | 286.90 | 31.67 |  |
| 8 | 31°33'2″ | 92°5'59″ | 559.20 | 3.55 | 35 | 31°25′38″ | 86°39′44″ | 308.70 | 32.12 |  |
| 9 | 31°4′51″ | 92°55′20″ | 401.50 | 17.52 | 36 | 31°54′16″ | 85°53′47″ | 329.60 | 25.75 |  |
| 10 | 32°13′33″ | 92°49′30″ | 573.90 | 16.67 | 37 | 32°4'7″ | 84°55'55″ | 310.40 | 13.94 |  |
| 11 | 30°58′55″ | 92°43′27″ | 669.20 | 19.18 | 38 | 32°14'3″ | 84°19'33″ | 275.60 | 11.42 |  |
| 12 | 31°34'9″ | 92°4'29″ | 533.00 | 3.60 | 39 | 31°18′18″ | 83°57′37″ | 291.60 | 21.25 |  |
| 13 | 31°50′40″ | 92°20′33″ | 602.70 | 19.00 | 40 | 32°16′14″ | 83°49′34″ | 243.60 | 16.72 |  |
| 14 | 32°15′40″ | 92°14′43″ | 530.10 | 6.52 | 41 | 32°10′14″ | 83°49′34″ | 281.70 | 16.68 |  |
| 15 | 32°18′15″ | 91°54′36″ | 494.40 | 1.14 | 42 | 32°8′1″ | 81°6′12″ | 233.90 | 37.93 |  |
| 16 | 31°44'10″ | 91°50'30″ | 507.90 | 6.90 | 43 | 32°38′6″ | 82°56′10″ | 253.90 | 37.98 |  |
| 17 | 32°19′29″ | 91°43′22″ | 489.00 | (3.26) | 44 | 31°28′21″ | 82°6′18″ | 350.80 | 40.91 |  |
| 18 | 31°5′2″ | 91°41′15″ | 533.60 | 19.60 | 45 | 32°29′17″ | 82°42′20″ | 237.20 | 37.16 |  |
| 19 | 31°29′32″ | 91°2′56″ | 437.90 | 15.04 | 46 | 31°17′32″ | 80°39′52″ | 236.80 | 33.48 |  |
| 20 | 31°26'24″ | 90°24'49″ | 395.50 | 11.66 | 47 | 31°52′39″ | 80°10′25″ | 245.60 | 26.20 |  |
| 21 | 31°25'24″ | 90°1'49″ | 367.50 | 5.56 | 48 | 32°15′37″ | 80°1′32″ | 184.80 | 20.29 |  |
| 22 | 31°35'24″ | 90°10'49″ | 399.50 | 8.64 | 49 | 33°34′21″ | 79°56′45″ | 309.50 | 51.62 |  |
| 23 | 31°28′13″ | 89°59′33″ | 379.60 | 6.05 | 50 | 32°43′6″ | 79°52′1″ | 284.20 | 21.13 |  |
| 24 | 31°22′53″ | 88°58′34″ | 312.70 | 18.32 | 51 | 33°38′16″ | 79°51′14″ | 312.30 | 55.34 |  |
| 25 | 31°27'35″ | 88°47'29″ | 290.90 | 17.94 | 52 | 32°52′19″ | 79°47′26″ | 250.60 | 25.19 |  |
| 26 | 30°57'35″ | 88°42'29″ | 323.80 | 8.90 | 53 | 33°15′39″ | 79°43′23″ | 292.00 | 39.27 |  |
| 27 | 30°55′59″ | 88°22′24″ | 366.40 | 12.21 | 54 | 33°28'48″ | 79°36'14″ | 340.80 | 49.84 |  |
| Note: MAT data were obtained from http://data.tpdc.ac.cn/zh-hans/data/ (Du and Yi, 2019).  The MAP was obtained from http://data.tpdc.ac.cn/zh-hans/data/ (Fang, 2019). | | | | | | | | | |  |
|  |  |  |  |  |  |  |  |  |  |  |

Table S2 The correlations between foliar C, N, P and soil nutrients in alpine meadow ecosystem

|  | C | N | P | SC | SN | SP | AN | AP |
| --- | --- | --- | --- | --- | --- | --- | --- | --- |
| C | 1.000 | 0.422 | 0.214 | -0.121 | 0.378 | 0.234 | 0.537^*^ | 0.421 |
| N |  | 1.000 | 0.831** | 0.215 | 0.391 | 0.308 | 0.406 | 0.428 |
| P |  |  | 1.000 | 0.289 | 0.394 | 0.372 | 0.421 | 0.430 |
| SC |  | - |  | 1.000 | 0.730^**^ | 0.734^**^ | 0.356 | 0.543^*^ |
| SN |  |  |  |  | 1.000 | 0.965^**^ | 0.515^*^ | 0.925^**^ |
| SP |  |  |  |  |  | 1.000 | 0.426 | 0.865^**^ |
| AN |  |  |  |  |  |  | 1.000 | 0.581^*^ |
| AP |  |  |  |  |  |  |  | 1.000 |
| Note：C: Leaf C concentrations; N: Leaf N concentrations; P: Leaf N concentrations;  SC: soil C concentrations; N: soil N concentrations; SP: soil P concentrations;  AN: soil available N concentrations; AP: soil available P concentrations; | | | | | | | | |

Table S3 The correlations between foliar C, N, P and soil nutrients in alpine steppe ecosystem

|  | C | N | P | SC | SN | SP | AN | AP |
| --- | --- | --- | --- | --- | --- | --- | --- | --- |
| C | 1.000 | -0.418 | 0.060 | -0.055 | 0.237 | -0.210 | 0.610* | -0.137 |
| N |  | 1.000 | 0.153 | 0.129 | 0.058 | 0.468* | -0.106 | 0.352 |
| P |  |  | 1.000 | -0.150 | -0.190 | -0.342 | 0.071 | 0.026 |
| SC |  |  |  | 1.000 | 0.940** | 0.729** | -0.176 | 0.796** |
| SN |  |  |  |  | 1.000 | 0.656** | -0.039 | 0.762** |
| SP |  |  |  |  |  | 1.000 | -0.187 | 0.821** |
| AN |  |  |  |  |  |  | 1.000 | -0.102 |
| AP |  |  |  |  |  |  |  | 1.000 |
| Note： C: Leaf C concentrations; N: Leaf N concentrations; P: Leaf N concentrations;  SC: soil C concentrations; N: soil N concentrations; SP: soil P concentrations;  AN: soil available N concentrations; AP: soil available P concentrations; | | | | | | | | |

Table S4 The correlations between foliar C, N, P and soil nutrients in desert steppe ecosystem

|  | C | N | P | SC | SN | SP | AN | AP |
| --- | --- | --- | --- | --- | --- | --- | --- | --- |
| C | 1.000 | -0.489 | -0.014 | 0.242 | 0.399 | -0.474 | 0.557* | -0.030 |
| N |  | 1.000 | 0.795** | -0.458 | -0.500* | 0.162 | -0.367 | -0.129 |
| P |  |  | 1.000 | -0.205 | -0.180 | -0.115 | 0.080 | -0.232 |
| SC |  | - |  | 1.000 | 0.915** | -0.346 | 0.797** | 0.149 |
| SN |  |  |  |  | 1.000 | -0.556* | 0.800** | -0.030 |
| SP |  |  |  |  |  | 1.000 | -0.647** | 0.645** |
| AN |  |  |  |  |  |  | 1.000 | -0.263 |
| AP |  |  |  |  |  |  |  | 1.000 |
| Note：C: Leaf C concentrations; N: Leaf N concentrations; P: Leaf N concentrations;  SC: soil C concentrations; N: soil N concentrations; SP: soil P concentrations;  AN: soil available N concentrations; AP: soil available P concentrations; | | | | | | | | |

| Table S5 The correlations between foliar N, P and climate factors | | | | |
| --- | --- | --- | --- | --- |
|  | N | P | MAP | MAT |
| N | 1 |  |  |  |
| P | 0.669^**^ | 1 |  |  |
| MAP | 0.145 | 0.343^*^ | 1 |  |
| MAT | 0.278^*^ | 0.002 | -.358^**^ | 1 |
